# Variation in developmental arrest among male orangutans: a comparison between a Sumatran and a Bornean population

**DOI:** 10.1186/1742-9994-10-12

**Published:** 2013-03-19

**Authors:** Lynda P Dunkel, Natasha Arora, Maria A van Noordwijk, Sri Suci Utami Atmoko, Angga Prathama Putra, Michael Krützen, Carel P van Schaik

**Affiliations:** 1 Anthropological Institute & Museum, University of Zurich, Winterthurerstrasse 190, Zürich, CH-8057, Switzerland; 2 Faculty of Biology, Universitas Nasional, Jalan Sawo Manila, Pejaten Pasar Minggu, Jakarta, 12520, Indonesia; 3 Faculty of Mathematics and Science, Universitas Indonesia, Depok, 16424, Indonesia

**Keywords:** Arrested development, Bimaturism, Bornean orangutan, Sumatran orangutan, Reproductive tactics

## Abstract

**Introduction:**

The presence of two sexually active male morphs with different reproductive tactics in a single species is rare among mammals. The most striking case of bimaturism among primates is exhibited by the orangutan (*Pongo spp*), in which one adult morph, the unflanged male, irreversibly develops into another one, the flanged form, but may remain arrested in the unflanged state for many years. However, it has been suggested that such arrest is less common among Bornean orangutans (*Pongo pygmaeus*) compared to Sumatrans (*Pongo abelii*). To investigate this possible inter-specific difference we compared both the number of developing males and the ratios of the two male morphs at two long-term study sites, Suaq Balimbing on Sumatra and Tuanan on Borneo.

**Results:**

First, we observed a significantly greater number of flanged than unflanged males per month in the Tuanan study area, whereas the opposite pattern held at Suaq. Second, the same contrast held for the total number of identified individuals over the study, with more flanged than unflanged males at Tuanan and the opposite at Suaq. These differences were mainly due to transient males. For Tuanan, the identification results were confirmed by detailed genetic analyses. Finally, we recorded a higher proportion of unflanged males that became flanged during any given year at Tuanan than at Suaq.

**Conclusion:**

These results show that developmental arrest is far more common at Suaq than at Tuanan. Preliminary comparisons suggest that this is a general contrast between the island taxa of orangutans and should help efforts to identify the function and proximate control of developmental arrest in orangutan males.

## Introduction

Sexual selection theory attributes major differences in mating behavior to the presence of alternative reproductive tactics (ART) [1-3]. ART can either be fixed over the lifetime, with individuals retaining a certain phenotype throughout life [3], or plastic, which allows a flexible response to the environment. Fixed ART, also called alternative strategies, reflect genetic polymorphism maintained by frequency-dependent selection and are thus characterized by equal average fitness. Examples include a marine male isopod (*Paracerceis sculpta*) with three different-sized male morphs [4,5], a live-bearing fish (*Poecilia parae*) with five distinct male color morphs [6] and the ruff (*Philomachus pugnax*), a sandpiper species with one conspicuous/colorful morph setting up a mating lek and an inconspicuous morph acquiring matings while being a satellite on such leks [7].

**Figure 1:**
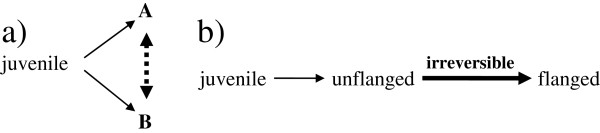
**Plastic alternative reproductive tactics. **(**a**) An individual reversibly develops into morph A or B (e.g. mandrills); (**b**) or an individual develops into morph A and then can irreversibly continue toward morph B, or remain in state A, as in orangutans.

The plastic tactics are adopted either in a reversible (Figure 1a) or a fixed (Figure 1b) sequence [1]. Irrespective of genotype, individuals adopt a tactic according to the current conditions or developmental state, but the tactics usually do not have equal fitness [3], as the less successful tactic is thought to make the best of a bad job. For instance, large males of the Italian tree frogs (*Hyla intermedia*) adopt the more successful calling tactic, whereas smaller males adopt the satellite tactic. Whenever the conditions allow it, a satellite frog can also employ the calling tactic, indicating that choice of tactic is reversible [8]. Among mammals, reversible intrasexual bimorphism is rare [9,10], but is found in some male primates. For instance, in mandrills (*Mandrillus sphinx*) the development of male adornments, especially the bright face and rump coloration and the size of testes, are sequentially reversible, and linked to dominance rank [11]. A similar phenomenon is the presence of clean and stained-chested males in Verreaux’s sifakas (*Propithecus verreauxi verreauxi*) [12].

Irreversible bimorphism (Figure 1b) is also found in some primates. Males go through a phase in which they have reached sexual maturity and are able to sire offspring, but have not yet acquired the complete set of secondary characteristics of fully mature males (e.g. [13]). This adult phase has traditionally, but misleadingly, been called ‘sub-adulthood’. In many species the time spent in this ‘sub-adult’ phase is variable. In male long-tailed macaques (*Macaca fascicularis*), for instance, variation in the timing of the transition into the mature morph with full secondary characteristics is linked to both a male’s intrinsic growth rates and the local social situation [14].

Among all primates, orangutans show the most flexible time span before attainment of the full set of secondary sexual characteristics, as suggested by early observations of female-sized male orangutans that were skeletally mature, with closed cranial sutures and mature dentition, but lacking cheek flanges, long fur, large body size, and an inflatable throat sac [15,16]. Such males are called unflanged, in contrast to the fully developed flanged males. This suspicion was confirmed by an observation from the wild [17], in which an unflanged male observed at Ketambe, Sumatra, only developed flanges twenty years after (genetically confirmed: [13]) siring of an offspring. Thus, he had undergone an extremely prolonged phase of arrested development, and must have been well over 30 years of age when developing flanges. In captivity, some Sumatran orangutans are known to grow flanges at the age of 11 years [18]. However, there are no known cases where a male, once flanged, reversed to the unflanged state.

The function of this unusual delay may be related to the differential mating strategies of the two morphs of sexually mature orangutan males [17]. Delgado and van Schaik [19] stressed the difference in social organization between Sumatran (*Pongo abelii*) and Bornean (*Pongo pygmaeus*) orangutans (see also 20), which are now considered separate species [20-24], and suggested that developmental arrest is more pronounced on Sumatra than on Borneo. The aim of this paper is to test this proposition. A difference in arrested development between the two islands would help to better understand the conditions in which this rare phenomenon could have arisen and to identify the proximate triggering mechanisms. Before we list our predictions we will first review the currently available information on mating strategies of the two species.

Unflanged males, both Sumatran and Bornean, look very similar to females, as they lack the secondary characteristics, but they have fully grown testes [25] and are able to sire offspring in captivity [26-28] and in the wild [13,29]. These males are highly mobile and actively search for females with whom they initiate matings and try to stay in association [30]. They have relatively high copulation rates and prefer to mate with fertile females [31]. However, based on the females’ preference for the dominant flanged male [13,32-34], unflanged males often have to resort to mating attempts with females even when the probability of fertilization is low [35]. Moreover, females often try to resist mating attempts by unflanged males, which results in forced matings [31,33,36-41]. This unusual combination of strongly resistant females and forced copulations is a reflection of female preferences [31,33].

On Sumatra, the local dominant flanged male attracts fertile females by emitting long calls [36]. His home range is smaller than that of other flanged males [42]. In general, sexually motivated females prefer the local dominant flanged male [13,32,33], seeking him out [30,43], and engaging with him in voluntary consortships, during which both the male and the female are likely to initiate mating. Flanged males never associate with each other, but when they meet, behave agonistically toward each other [42]. Dominant flanged males are able to sustain lengthy consortships, lasting weeks, in which they have a virtual monopoly on matings with the estrous female, suggesting that the paternity rate of non-dominant flanged males is virtually zero [44]. The main reason for this is that if non-dominant males emit long calls they will not only attract females but also the dominant male and thus are likely to be chased away. On the other hand, not calling is not a promising option either, since flanged males are not as mobile as unflanged males [45] and thus cannot employ their tactic of actively searching for females.

On Sumatra, females with offspring tend to range within earshot of flanged males, that is a female stays at a distance at which she can hear a vocalizing dominant male, possibly to avoid harassments by unflanged males [43,46]. However, tolerance of unflanged males by flanged males engaged in a consortship with fertile females is commonly observed [22,25,35,47], probably because the faster unflanged males can escape when attacked by flanged males. Since paternity results are still scarce [13,29], it is not quite clear what the reproductive success of each kind of male tactic is under various social or demographic conditions. However, it has been suggested that unflanged males are more successful with the less attractive nulliparous females and during periods of unstable dominance relations among flanged males, when females are not effectively mate-guarded [13,48].

As a possible explanation for the potentially long developmental arrest of unflanged male Sumatran orangutans, Utami et al. [48] therefore suggested that the highly mobile unflanged males are at least somewhat reproductively successful when females are not in association with the dominant flanged male, whereas subordinate flanged males’ reproductive success is expected to be close to zero [19]. Thus, unflanged males probably have lower per capita success than the dominant flanged male, but higher success than subordinate flanged males [48]. Therefore, males may benefit from remaining unflanged until they can become the locally dominant flanged male.

On Borneo, flanged males seem to be more escalation-prone, in that they engage more often in physical fights [42,49], and dominance relations among them appear less stable compared to Sumatra [38,49,50]. Bornean flanged males, even dominant ones, engage in shorter consortships with females than the Sumatrans [32,38], and also force matings. Moreover, females have been observed to copulate with multiple flanged males within a period of several weeks [35,38]. On the other hand, Bornean flanged males tend to travel much more on the ground, which makes them more mobile and able to locate and actively follow and displace the more arboreal unflanged males from females. Bornean females, unlike Sumatran ones, do not engage in earshot association with flanged males [51]. On the whole, then, non-dominant flanged males on Borneo are likely to be more successful reproductively, both in absolute terms and relative to unflanged males, than their Sumatran counterparts. Consequently, arrested development may be relaxed or absent among them.

The proposal that arrested development may be more pronounced on Sumatra than on Borneo [19] has never been tested in detail. To test it we will examine the following predictions. First, we expect relatively more flanged males in a Bornean population if males are less likely to arrest their development on Borneo than on Sumatra (alternative explanations for different morph ratios will be examined in the discussion section). Second, if this difference in male morph ratios is due to differences in developmental arrest, we expect that the probability that a male grows flanges during a certain period of time will be higher on Borneo than on Sumatra [44]. The third prediction concerns the behavioral differences between resident and transient males [36,37,41,52]. Transients largely include individuals who use the area to pass through one time, and never come back, or individuals with very large home ranges, who occasionally appear, during periods of local food abundance or during periods when local females are reproductively attractive [38]. On Sumatra, the males able to break the monopoly of the dominant flanged males during such periods are the unflanged ones [42], whereas on Borneo multiple flanged males but few unflanged males mate with fertile females [Dunkel, personal communication]. Thus, we predict a higher number of transient unflanged males on Sumatra and a higher number of transient flanged males on Borneo.

In this study, we took great care to identify individual males to obtain the most accurate estimate of their number in the population, by using careful descriptions, photographic records and genetic confirmation of identity, at least for one site.

## Results

### Numbers of flanged versus unflanged males

#### Monthly male presence

**Figure 2:**
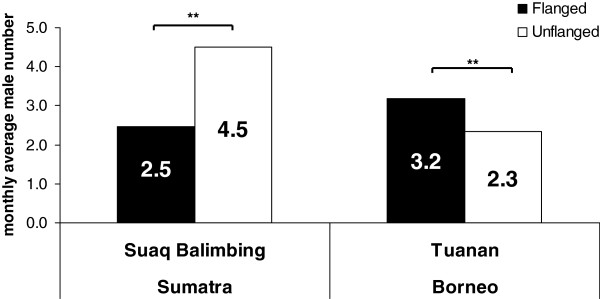
**Monthly male presence.** The average monthly number of identified flanged (black) and unflanged (white) males in Suaq (Sumatra) and Tuanan (Borneo).

At Suaq (Sumatra), the mean number of flanged males recorded in the study area in any given month during the 59-month observation period was 2.5, and that of unflanged males 4.5 (Figure 2); this difference was significant (Mann–Whitney U = 996.5; N = 59 months; P < 0.001, 1-tailed). At Tuanan (Borneo), however, we found the opposite, as significantly more flanged than unflanged males were recorded (3.2 flanged versus 2.3 unflanged males, Mann–Whitney U = 1894.5 N = 72 months; P = 0.002, 1-tailed).

#### Total number of identified individuals

**Figure 3:**
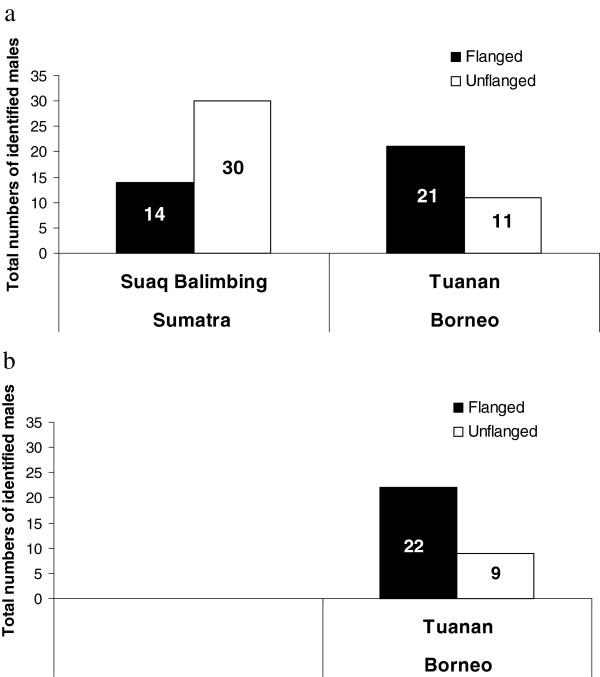
**Total number of identified individuals.** (**a**) The total number of identified flanged males (black bars) in Suaq Balimbing and Tuanan, as compared with the total number of unflanged males at the same sites (white bars), based on field identifications. (**b**) The total number of genetically identified flanged (black bar) and unflanged (white bar) males, for Tuanan only (unknowns genetically identified as new individuals are not included).

The total number of flanged versus unflanged males in Suaq identified in the field was 14 vs. 30, while in Tuanan it was 21 vs. 11 (Figure 3a). Thus, we found, based on comparisons of detailed descriptions and photographs, that the Suaq population was biased toward unflanged males, whereas the Tuanan population was biased toward flanged males. This difference between the male proportions at the two sites was highly significant (Pearson’s Chi-Square Exact Sig. 1-sided: *X*^2^ = 8.523, df = 1, N = 76, P = 0.003).

For the Tuanan population, we could use genetic analysis to evaluate the accuracy of the procedure applied at both Suaq and Tuanan. There were three categories of potential errors (Table 1). First, in the field animals were split conservatively. In the absence of genetic analysis, they would have been lumped again in the final tally. Indeed, genetic analysis confirmed all six to be the same individual (four flanged and two unflanged, see Table 1), thus confirming the normal procedure. Second, genetic analysis showed that three of the individuals split in the field were actually of the same individual. Thus, we had overestimated the total number of males by three. Third, on the other hand we had wrongly lumped two individuals in the field that upon genetic analysis turned out to be distinct individuals. In total, then, we had overestimated the number of unflanged males by two and underestimated the number of flanged males by one (Table 1 and Figure 3b). Because the errors revealed by genetic analysis showed that the estimated flanged/unflanged male ratio at Tuanan was conservative relative to the difference with Suaq as estimated through normal descriptions, our earlier conclusion was confirmed by this analysis.

**Table 1:** Identification errors made in the field affecting the total number of individuals in Tuanan

	**Flanged males**	**Unflanged males**
Visually identified and confirmed by genetic analysis	20	9
Conservatively split in the field, genetically confirmed one individual	4	2
Incorrectly split in the field, genetically shown to be the same individual	1	2
Wrongly lumped in the field, genetically shown to be separate individuals	2	0
Total balance	Underestimated by 1	Overestimated by 2

In addition, we collected several samples of unnamed individuals that would not have been included in a normal tally. Among the numerous unknowns (over 20) we found three more new unnamed individuals, one flanged and two unflanged. This indicates that a certain proportion of unidentified individuals in the field are indeed novel individuals. However, since they remained unknown these are probably transient individuals.

### Rates of flange development at Suaq and Tuanan

At Suaq, 16 different unflanged males were included in the sample, accounting for a total of 58 unflanged male years. In this dataset, we recorded only a single male developing flanges resulting in an estimated annual probability of 0.017, or approximately once every 58 years. At Tuanan, 8 different unflanged males were included in the sample, totaling 35 unflanged male years. We recorded 4 cases of flange development, for an estimated annual probability of 0.114, or once every 9 years. The males concerned had been encountered both as unflanged and subsequently as flanged males, and their identities were genetically confirmed. Despite the small sample size, the difference between the two proportions (1/58, 4/35) approaches significance (Fisher’s exact test P = 0.08; see also discussion). The nearly seven-fold difference observed strongly suggests that there is a large between-site difference in the probability that an unflanged male grows flanges and other secondary sexual characteristics in a particular year.

### Numbers of resident and transient males based on their monthly presence

**Figure 4:**
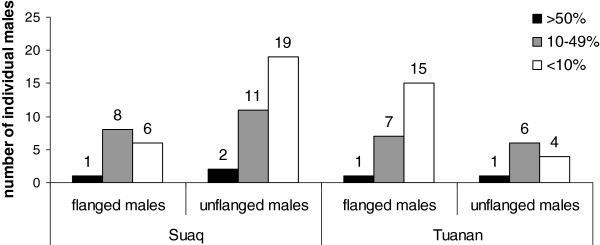
**Numbers of resident and transient males based on their monthly presence.** The number of individuals included into three different monthly presence categories. Black: monthly presence > 50%, grey: monthly presence 10 until 49% and white: monthly presence < 10%.

The total numbers of true residents (defined as being present > 50% of the months) and partial residents (with a 10-49% monthly presence) among the flanged and unflanged males during the study period (72 months for Tuanan and 59 for Suaq) did not significantly differ between the two study sites (sample size for true residents too small for statistics; for partial residents: Pearson’s Chi-Square exact 2 sided: X^2^ = 0.427, df = 1, N = 32, P = 0.720) (Figure 4). However, for the number of transients (males seen less than 10% of the months) we found significantly more transient unflanged males in Suaq and more transient flanged males in Tuanan (Pearson’s Chi-Square exact 2 sided: X^2^ = 13.065, df = 1, N = 44, P =0.001).

## Discussion

All three basic sets of results (the monthly presence in study area, the total number of males identified, and the observed rate of flange development) strongly suggest that males at Tuanan (Borneo) experience far less developmental arrest then males at Suaq (Sumatra). The consistency of these results suggests that errors are not responsible for this pattern.

One may of course question the accuracy of the estimate of 0.017 flanging events per year for Suaq. However, in contrast to Tuanan, there is also only one known record of a male with partial flanges in Suaq, despite comparable observation effort. This single record mirrors that made at Ketambe, also on Sumatra, by te Boekhorst et al. [53]. In their study, no flanging events were recorded in 61 unflanged male years (the flanging event reported by Utami et al. [48,54] occurred later). In contrast, the Tuanan flanging events were striking, and would also have been noted without genetic analysis. We encountered 5 partially flanged males in Tuanan, who could easily be distinguished from males with shriveled flanges due to old age or poor body condition in the event of food scarcity (although we cannot exclude that some males may go through flange development very slowly). Thus, the rate of flange development at Tuanan was many times higher than at Suaq and Ketambe.

The extremely low rate observed at Suaq and Ketambe may indicate that many unflanged males on Sumatra may never grow flanges at all, and in fact may remain arrested for their entire life. Alternatively, it could indicate that flange development occurs in synchronized bursts. However, both known cases, one at Suaq and one at Ketambe, were isolated cases. At Ketambe, for instance, the unflanged male grew flanges after the take-over of the dominant male by an intruding male, and subsequently challenged the intruder when he had become flanged [54].

The consistency of our results could be questioned because of discrepancies between the flanged / unflanged proportions in the monthly presence data and the total number of identified individuals. The monthly presence data could have been biased. For Suaq, the flanged / unflanged ratio in the monthly presence records of 0.6 is quite close to the proportion of their total identified numbers. For Tuanan, in contrast, the ratio for the number of total identified individuals (1.9) is higher than the monthly presence ratio (1.4). This discrepancy can be explained by the fact that most of the flanged males at Tuanan are transients, that is, males without a fixed home range but roving around. In contrast, unflanged males made up the highest number of transients in Suaq (Figure 4).

This study was the first to incorporate genetic analyses to identify all wild orangutans in the study area at Tuanan. The total numbers of flanged and unflanged males estimated through field observations and genetic identity analyses did not vary substantially. Possible errors of identification in the field, like unintentionally lumping of two different individuals into one or splitting of a single individual into two, could be detected in Tuanan with genetic analyses. The results, however, revealed that such errors were minor (see Table 1). Thus, although the application of genetic techniques did improve the accuracy of identification procedures at Tuanan, the differences were so minor that they do not compel us to revise the conclusion that there are major differences between Suaq and Tuanan in male developmental arrest. Nevertheless, orangutan researchers should be aware of these problems and put efforts into correct field identification.

### Alternative explanations

As this was an observational study, we should not rule out other possible explanations for the different male morph ratios at the two study sites. There are two issues here: Estimated rates of flange development and morph ratios. The rates of flange development for two Sumatran sites, Suaq and Ketambe [53] were made in different periods and covered multiple years. They are therefore likely to be reliable. The one for Tuanan is only based on one site and a relatively short period compared to adult male lifespan. It could be artificially high, but we could not construct a realistic scenario that could bring this about. The island differences in morph ratios are found across many sites and at Ketambe, for instance, have remained stable over decades. Moreover the observations at both our study sites, continued beyond the periods compared here (even though observations in Suaq were only resumed in 2007), allowing us to compare the numbers for the more recent situation. Data collected in Suaq 2007 until 2011 (E. Meulman, pers. comm.) and the additional data from Tuanan after 2009 (B.Spillmann, pers. comm.) show the same pattern as the larger dataset analysed here.

Another cause of modified morph ratios could be selective mortality of one age class due to external disturbance, especially logging. In general, mortality among orangutans is extremely low [55]. In Ketambe and Suaq, there was no logging in the study area during decades leading up the study. Some logging elsewhere could have led to male influxes, but there is no reason to expect flanged and unflanged males to differ. Tuanan has indeed been subject to selective logging in the late 80s-early 90s. If logging causes high infant mortality this would have led to a gap in the cohort that was born before and during the logging period, which would account for the relatively low number of unflanged males. However, Bornean orangutans seem to be less affected by logging than Sumatrans [56-58]. Moreover, morph ratios biased toward flanged males have also been recorded in study areas that were never logged, or in the 70s before logging [36,38,39,59]. Finally past logging would not explain the higher rates of flanged development in Tuanan compared to Suaq. We can therefore exclude logging as a general cause for possible island differences in morph ratios. Furthermore we can exclude differences in dispersal between populations of the two islands as recent studies convincingly showed that on both islands males are the dispersing sex, whereas females are philopatric [24,29,60-62].

Finally, we can exclude hunting as the cause of the higher proportion of flanged males in Tuanan. Hunting is absent in the region of Suaq and Ketambe [63], but is found patchily on Borneo. However, because flanged males are bolder and more likely to come close to humans, especially as crop-raiders, they are more likely to get killed. Thus, differential hunting can also not account for the differences in morph ratios.

### Inter-site differences or inter-island differences?

Does the difference in flanged /unflanged male ratios found in this study generalize to a difference between Sumatra and Borneo? A previous review [19] suggested this based on the number of flanged and unflanged males reported in papers on orangutan behavior. More flanged than unflanged males are mentioned in reports for the Bornean study sites Mentoko [40], Tanjung Puting [38,39], Kinabatangan [29] and Gunung Palung [64], and fewer flanged than unflanged males for the Sumatran site Ketambe, which is about 70 km from Suaq [51], but also for the Bornean site Sabangau [49]. These reports provide the numbers included for particular analyses and not necessarily the total number of identified individuals, let alone the total number of males visiting a study site. It should be noted that the largest inter-morph difference occurs among transient males, which may not always have been equally recorded in all studies. It is also possible that Bornean sites occasionally have periods in which one flanged male is clearly dominant, leading other flanged males to avoid the area. Nevertheless, it is unlikely that the general difference between the Sumatran and Bornean sites is completely due to methodological details across sites. Moreover, the average monthly number of the two male morphs at Ketambe showed the same trend as Suaq [42]. Finally, te Boekhorst et al. [53] also found very low rates of flange development at Ketambe (none in 61 unflanged male years involving 13 different males over a 12-year period). If the males at Ketambe and Suaq follow the same developmental tactic we can lump the data on flanging rates. The difference in flanging rate between Tuanan and the two Sumatran sites (Ketambe and Suaq) combined becomes significant (Fisher’s exact test P = 0.013). Overall, these data suggest that the Tuanan-Suaq difference reflects a more general island difference, but more detailed future work at other Bornean sites is needed.

The outcome of this study may have implications for our understanding of the proximate function and the regulation of developmental arrest in orangutan males. As to the ultimate reasons for the differences between the two orangutan species, Pradhan et al. [65] developed a model that points to the sexual monopolization potential of dominant flanged males as the key factor for differences in arrested development. Several studies already found that on Sumatra consortships between flanged males and females last longer than on Borneo [32,38,44], suggesting a much higher monopolization by dominant flanged males on Sumatra at the expense of all other flanged males. Therefore, subordinate flanged males’ access to females is near-zero and lower than that of unflanged males, who do occasionally get matings with fertile females and mate frequently with nulliparous females [48]. On Sumatra, the prolonged arrested development may therefore be adaptive. On Borneo, however, the monopolization potential of a single dominant flanged male is low and other less dominant flanged males also have access to females. But because flanged males are always dominant to unflanged males, the latter can be displaced easily and probably have lower reproductive success. In future work, we will test this hypothesis in more detail with data from Suaq and Tuanan.

As to the proximate regulation, studies in captivity and in the field have shown that arrested males have lower testosterone and dihydro-testosterone levels than developing and flanged males, but they have enough testicular steroids to support sexual function and fertility [26,66,67]. However, the conditions that elicit the rise in testosterone in developing males remain unclear. Some captive studies suggested that the presence of a flanged male, and therefore socio-endocrine effects of social interactions, could be the proximate reason for the arrest of unflanged males [26-28,68,69]. However, this is unlikely in the wild. First, multiple flanged males are always present at a site [48]. Second, less pronounced or absent developmental arrest, as in Tuanan, produces higher absolute numbers of flanged males (ceteris paribus). Instead we suggest that the increased access to potentially fertile females by unflanged males, especially during periods of unstable dominance relations, may trigger the development of secondary characteristics [53]. On Borneo, however, dominance ranks are unstable most of the time, which allows less dominant flanged males access to females. As yet, however, this hypothesis remains untested.

## Conclusion

Male developmental arrest is quite rare among mammals. The unexpected inter-island differences in orangutans documented here provide us with an unprecedented opportunity to examine both the conditions in which this adaptation could evolve and identify the proximate triggering mechanisms. It would be especially interesting to examine the flexibility in developmental arrest among Bornean males in more detail to assess whether some males are capable of showing long-term arrest, as on Sumatra.

## Material and methods

### Study sites and animal sampling methods

Data were used from two study sites: Suaq Balimbing on Sumatra and Tuanan on Borneo. The Sumatran data were collected over 59 months from 1993–1998 at the Suaq Balimbing research station (ca 5.5 km^2^; 3°04’N, 97° 26’E), Gunung Leuser National Park. The local orangutan population density was 6.9 ind/km^2^[70]. This site is located at near-sea level and primarily consists of swamp forest on shallow peat. Most of the study area was pristine, but nearby areas were subject to selective logging.

The data on Bornean orangutans were collected over 72 months from June 2003 until mid 2010 at the field station Tuanan (ca. 7.5 km^2^, 2° 09’S; 114° 26’E) inside the Mawas Reserve in Central Kalimantan. The area supports an average orangutan density of 4.25 ind/km^2^[71], and consists of peat swamp forest on shallow peat, also at near sea level. It had been subject to selective commercial logging in the late 80s and early 90s, followed by additional opportunistic logging.

Orangutans at both study sites were followed from morning nest to evening nest, using standardized focal animal sampling techniques described in detail at the orangutan network website [72] of the Anthropological Institute and Museum, University of Zurich. Individuals on which behavioral data were collected were called focal animals, whereas individuals spotted in the forest, but without taking data on them were the non-focals.

To estimate the exact number of individual males in an area is not a trivial task, since the total number of individuals encountered in an orangutan study site is subject to continuous change, for two reasons. First, unlike virtually all other primates, orangutans do not live in distinct groups or communities [51,73]. Second, both unflanged and flanged males usually range widely, and because study areas are necessarily less than 1000 hectares, come and go [42]. As a result, new individuals may appear at all times, and orangutan identification is not straightforward. Because the new ones are usually not habituated, it may be difficult to visually identify them.

The main method for identifying individuals was based on comparisons of descriptions and standardized sketches complemented by lists of peculiarities, such as stiff fingers and scars, and photographs. However, this method is subject to errors, especially for males, due to incorrectly splitting identical males or incorrectly lumping different males. Most studies applied the following procedure: When in doubt, males were given distinct names to be able at some later moment to either keep them separate or lump them again depending on further evidence. Without further evidence, however, such provisionally separate males would be lumped again at the end of the study.

During the last decade, one additional technique has become available that for the first time makes it possible to estimate the magnitude of these errors. Genetic analysis of individual identities, based on non-invasively collected fecal samples, became the tool of choice for individual identification in situations where field methods did not give unambiguous answers. For both field sites, identification was done with help of descriptions and photography comparisons, but for only Tuanan we additionally collected fecal samples for later genetic analyses.

We followed two procedures to identify the ratio of flanged to unflanged males in the population. First, we counted the number of each class observed in the study area each month, either as focal animals or in association with focal individuals, or encountered during other activities in the forest. This monthly number probably depends on the hours spent in the forest, yet there is no reason to assume a bias toward flanged or unflanged males with differential research effort.

A bias could arise when one class of adult males is far more likely to be transient. Hence, second, we also counted the total number of identified individuals of each class. (In Tuanan, three developing males were included as unflanged males and one as flanged and in Suaq, one as flanged, depending on the timing of their flange development). The latter is also reported by most other studies.

Although the second measure is intuitively obvious, there are various reasons to prefer the first one. First, it is less sensitive to uncertainties in identification (a male is added as present even if he remains unidentified), and it is less likely that multiple unidentified males of the same morph visit a study area during a single month than in a period covering multiple years. Moreover, these numbers are a direct reflection of the actual number of males of each morph competing at one time.

To estimate male presence in the area, we determined the monthly presence for all individuals. Thus, each identified male recorded in the study area during a particular month was included into the monthly presence file. Subsequently, the males were placed into one of three different presence categories. True residents were defined to be present at least 50% of the months, and thought to include the study site in their core area. Males with lower presence scores were divided into two classes. Partial residents were males with a monthly presence of 10 to 50%. They were probably nearby residents, who had their core area outside but near the study site and were attracted either by periods of high food abundance or by sexually attractive females. We defined transients as males with a monthly presence of less than 10%. They were observed to visit the study area only once or rarely, and were generally seen to pass through.

To estimate the likelihood that a male grows flanges, we determined the number of “unflanged male months”. Males entered the sample in the year of their first observation in the study area and were eliminated from it upon the time they grew flanges or after the year of their last recorded presence. Only males with more than 10% monthly presence and sighted in at least two different years were included into the sample.

All statistical analyses were done in SPSS 14.0.

### Genetic sampling and analysis for Tuanan

When possible, one or more fecal samples were collected whenever an orangutan with uncertain identification was encountered. Sample collection was carried out as described in the Genetic Sampling Protocol from the Anthropological Institute and Museum, University of Zurich [74]. For details on the genetic analyses of these samples see [60].

Individuals were genotyped at 6 nuclear microsatellite markers and subjected to identity analyses using Cervus 3.0 [75]. The six markers had a combined non-exclusion probability of 1.36 × 10^-5^ and 8.90 × 10^-3^ for unrelated individuals and full siblings, respectively, suggesting that samples with different genotypes were indeed from different individuals.

## Competing interests

The authors declare that they have no competing interests.

## Authors’ contributions

LPD collected data on wild orangutans for a total of 32 months at the Tuanan station including fecal samples in Central Kalimantan, analyzed data and drafted the paper. NA and MK performed the genetic analyses. APP assisted in data collection. CPvS and MAvN conceived the study. CPvS, SSUA and MAvN coordinated data collection and management in Suaq and Tuanan. LPD and CPvS wrote, and all authors read and approved, the manuscript.
